# Spinal Epidural Abscess: Raising the Index of Suspicion

**DOI:** 10.7759/cureus.85465

**Published:** 2025-06-06

**Authors:** Daniel Prabahkar, Iqra Qazi, Sunita Devi, Samar Eldahtoury, Aamer W Janjua

**Affiliations:** 1 Department of Medicine, Sam Houston State University College of Osteopathic Medicine, Conroe, USA; 2 Department of Medicine, Baptist Hospitals of Southeast Texas, Beaumont, USA

**Keywords:** atypical symptoms, delayed diagnosis, methicillin-sensitive staphylococcus aureus (mssa), peripheral neuropathy, spinal epidural abscess, spinal infections

## Abstract

A spinal epidural abscess is an uncommon but life-threatening infection. Typically, it arises secondary to an insult that provides bacterial entry into the bloodstream. Often, spinal epidural abscesses are managed with a combination of surgical intervention and systemic antibiotics in order to minimize the risk of antibiotic resistance and recurrence. The standard of diagnosis for spinal epidural abscesses is through contrast-enhanced magnetic resonance imaging (MRI), which has high sensitivity and specificity. This particular case describes an indolent course of a spinal epidural abscess that presented with neurological deficits unaccounted for by preliminary spinal imaging, underscoring the value of clinical reasoning in patient assessment. An 81-year-old Caucasian female was brought to the emergency department by her daughter with concern of low back pain and a 12-hour history of weakness in the bilateral lower extremities. Her lumbar pain worsened with spinal extension and improved with spinal flexion. She denied any injury, fever, or bowel and bladder incontinence. A review of her past surgical history revealed a recent left shoulder arthroplasty complicated by postoperative cellulitis. She denied any alcohol, tobacco, or drug use. At presentation, her respiratory rate was 28 breaths/minute, and all other vital signs were within normal limits. A physical exam demonstrated bilateral lower extremity strength 3/5, straight leg raising test positive on the right, and the remainder of the exam was unremarkable. To investigate mechanical spinal cord compression, an MRI without contrast of the lumbar spine was obtained. This revealed chronic degenerative changes and failed to explain her acute onset of weakness. Complete blood count revealed a white blood cell count of 16.0 x10^3^/µL (reference range: 4.5-11.5 x10^3^/µL), and in correlation with her tachypnea, bacteremia was now suspected. Empiric antibiotic treatment was promptly started; however, despite intervention, she failed to show signs of improvement, and blood cultures were inconclusive. Finally, on day three of admission, re-evaluation of her neurological deficits demonstrated +1 patellar reflexes in both legs. Given her bilateral lower extremity weakness, leukocytosis, and low back pain, the index of suspicion for a spinal epidural abscess increased, and she underwent a contrast-enhanced MRI of the lumbar spine. The imaging revealed a 17.1 mm abscess. Intravenous nafcillin was started, and she was transferred to a higher level of care for neurosurgical evaluation. There, she underwent emergent surgical debridement of the abscess and was discharged with a six-week course of outpatient intravenous oxacillin infusions. In summary, by assessing the entire clinical picture and not dismissing her persistent symptoms in spite of equivocal MRI findings, the abscess was correctly identified. Delays in the diagnosis of spinal epidural abscesses occur far too frequently in clinical practice. Therefore, this case reflects on the importance of systematic clinical reasoning, a thorough neurological exam, vigilance against anchoring bias, and considering a broad differential to minimize diagnostic delay and improve patient outcomes.

## Introduction

Spinal epidural abscesses are considered a life-threatening emergency; patients may rapidly deteriorate and suffer compromised neurological function [1,2]. The incidence of this diagnosis is estimated to be between two to eight cases per 10,000 hospital admissions, where 11-75% of cases are initially misdiagnosed. The mean age of diagnosis is 57.2 years and is associated with intravenous drug use and diabetes mellitus. Prognosis can be grave, with 4-22% of cases resulting in irreversible paralysis and 5% of cases resulting in death from sepsis [3]. While the variety of published case reports on this topic is limited, this case is unique in that the progression of symptoms was gradual and features of spinal cord compression preceded any evidence of infection, allowing this diagnosis to imitate other myelopathies.

Typically, what is described in the literature for spinal epidural abscesses is a triad of symptoms: fever, back pain, and neurological symptoms [4]. While this triad is specific, it lacks sensitivity, meaning many cases may not present with all three symptoms [5,6]. In such examples, early diagnostic blood cultures and imaging findings were critical for diagnosis even without the overt triad of symptoms [7-9]. Currently, the standard for diagnosis is contrast-enhanced magnetic resonance imaging (MRI), which has the greatest accuracy [10]. Another benefit of this imaging modality is its high sensitivity for detecting spinal epidural abscesses by the presence of paraspinal edema [11].

However, therein lies the diagnostic challenge; when imaging fails to detect an abscess and clinical features are initially unremarkable, a spinal epidural abscess may go undetected. Therefore, this case demonstrates how clinical reasoning, a thorough neurological exam, and a high index of suspicion can be applied to correctly identify this disease among the causes of neurological deficits.

## Case presentation

An 81-year-old Caucasian female is accompanied by her daughter to the emergency department with concern of sudden-onset weakness in her legs. Her symptoms began earlier that morning without progression. Although she was able to move her limbs, her concern was that she could not bear weight on her legs. Associated with her weakness was a dull pain in the lower back, which she described as moderately severe and occasionally radiating through the right leg. Spinal flexion improved the pain, and spinal extension worsened it. She denied any trauma to the back and denied symptoms of generalized fatigue, fever, headache, lightheadedness, palpitations, shortness of breath, nausea, vomiting, diarrhea, bowel or bladder incontinence, numbness, or upper extremity weakness. In contrast, she reported improving dysuria, as approximately one week earlier, she was diagnosed with an uncomplicated cystitis. She delayed filling her prescription for nitrofurantoin and was now only on her third day of taking the medication. Further review of her past medical history revealed hypertension, type 2 diabetes mellitus, hypothyroidism, congestive heart failure, and atrial fibrillation status-post pacemaker placement. In regard to these diagnoses, she reported adherence to her home medications. Her previous surgeries were notable for a left humeral surgical fixation four months earlier, which was complicated by cellulitis, as well as bilateral total knee arthroplasties performed years prior. She denied any alcohol, tobacco, or drug use. At home, she lived with her daughter and remained typically independent with her activities of daily living.

Her bilateral lower extremity weakness and right leg pain with lumbar extension raise the concern for etiologies that limit the tolerance for foraminal space narrowing. For example, conditions such as disc herniation, spinal stenosis, and degenerative disc disease. Additionally, the sudden onset of lower extremity weakness following the initiation of a new medication raises the possibility of a nitrofurantoin-induced myopathy. Similarly, her weakness may also be secondary to failed treatment of her urinary tract infection and the progression of pyelonephritis and urosepsis. Other differential diagnoses to consider include spondylolisthesis, spinal epidural abscess, and malignancy. All of which could lead to spinal cord compression and contribute to her limb weakness. Furthermore, the bilateral nature of the weakness expands the differential to include neurological conditions such as multiple sclerosis, Guillain-Barré syndrome, myasthenia gravis, Lambert-Eaton myasthenic syndrome, diabetic neuropathy, and subacute combined degeneration.

**Figure 1:**
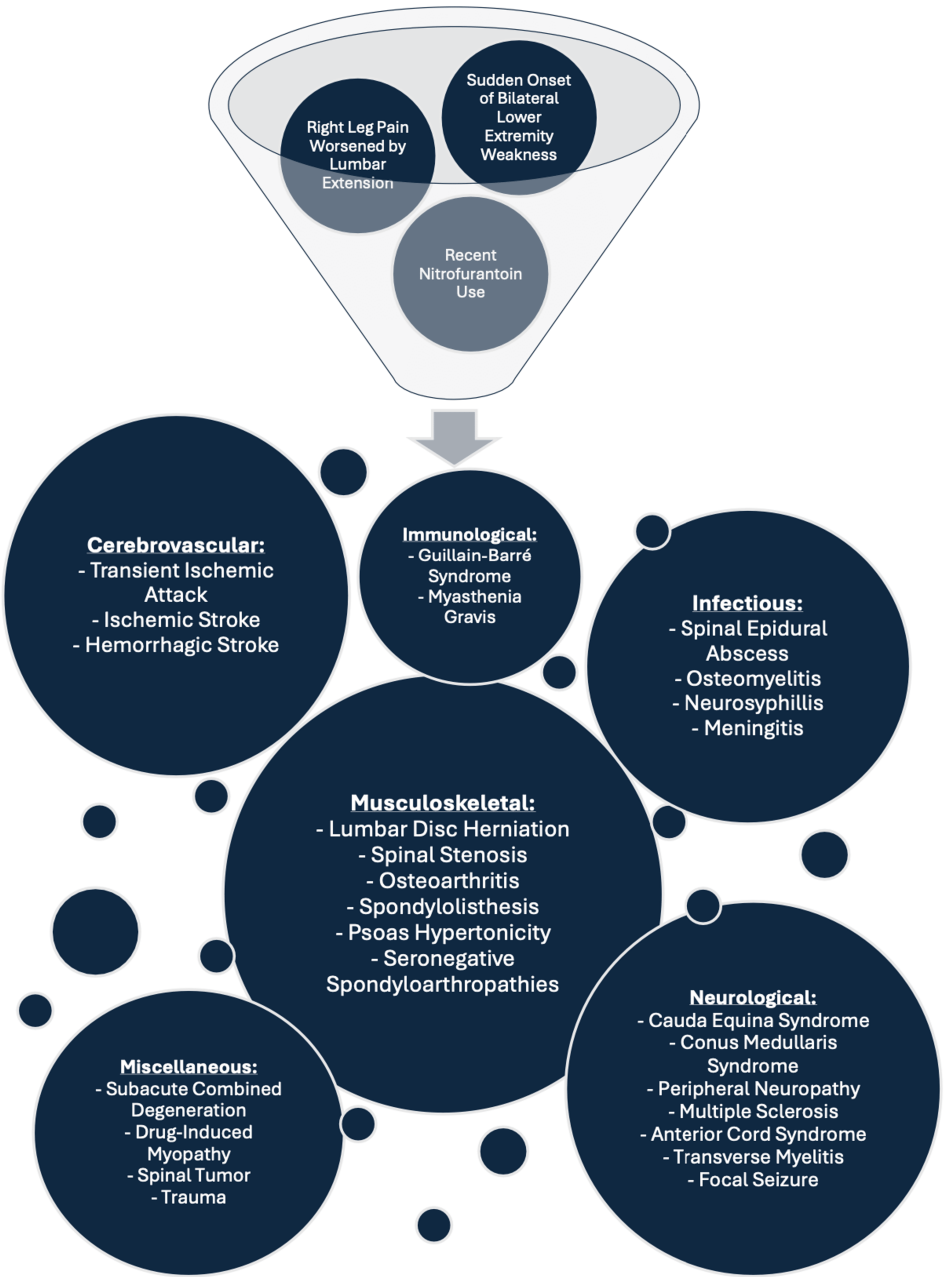
Visual representation of the differential diagnoses

At this stage in a patient's care, it is crucial for clinicians to avoid anchoring to an initial diagnosis. A thorough work-up is necessary to rule out a broad range of potentially life-threatening conditions that present with lower extremity weakness (Figure 1).

In the emergency department, her heart rate was 89 beats/minute, blood pressure 119/71 mmHg, respiratory rate 28 breaths/minute, temperature 98.9°F, and oxygen saturation 96%. A physical exam revealed pupils were equal, round, and reactive to light. Extraocular movements were intact. Cranial nerves were 2-12 intact, and there was no facial asymmetry. Inspection of the nasal passages revealed moist mucous membranes and a clear oropharynx without erythema or tonsillar edema. Cardiac exam revealed a regular rhythm without murmurs, rubs, or gallops to auscultation and no jugular venous distension. Lung fields were clear with vesicular breath sounds bilaterally. The abdomen was soft, nontender, and bowel sounds were normoactive. Posteriorly, no costovertebral angle tenderness. Genitourinary exam was without lesions, sores, or suprapubic tenderness. Upper extremity strength was 5/5, lower extremity strength 3/5, deep tendon reflexes +2 in all extremities, no saddle anesthesia, no Hoffmann sign, no dysdiadochokinesia, and no clonus. The straight leg raising test was positive on the right at less than 70° hip flexion.

Several diagnostic tests were also performed. Notably, laboratory results demonstrated an elevated white blood cell count of 16.0 x10^3^/µL (reference range: 4.5-11.0 x10^3^/µL). By coordinating this finding with her tachypnea, she met the diagnostic criteria for systemic inflammatory response syndrome (SIRS). Thus, blood cultures were promptly obtained from multiple sites, and she was initiated on empiric antibiotic treatment for sepsis. Measured inflammatory markers are described in Table 1. Despite these laboratory findings, her pulmonary, abdominal, and pelvic exam, along with urinalysis and chest X-ray, did not reveal a clear source of infection. CT scan of the abdomen and pelvis without contrast also revealed no acute changes. However, a CT scan of the lumbar spine without contrast did visualize degenerative disc disease, posterior disc bulge at L3-L4, foraminal stenosis, and central stenosis.

**Table 1:** Pertinent complete blood count values WBC - white blood cell count; NEUT - neutrophil; LYMPH lymphocyte; MONO - monocyte;  EOS - eosinophil; BASO - basophil; ABS NEUT - absolute neutrophil count

Lab Test	Patient value	Female reference range
WBC	16.0 ×10³/µL	4.0-10.0 ×10³/µL
NEUT %	86.70%	50-60%
LYMPH %	6.70%	20-50%
MONO %	5.40%	2-8%
EOS %	0.10%	1-4%
BASO %	0.20%	0.5-1%
ABS NEUT	13.9 ×10³/µL	2.5-7.0 ×10³/µL

**Figure 2:**
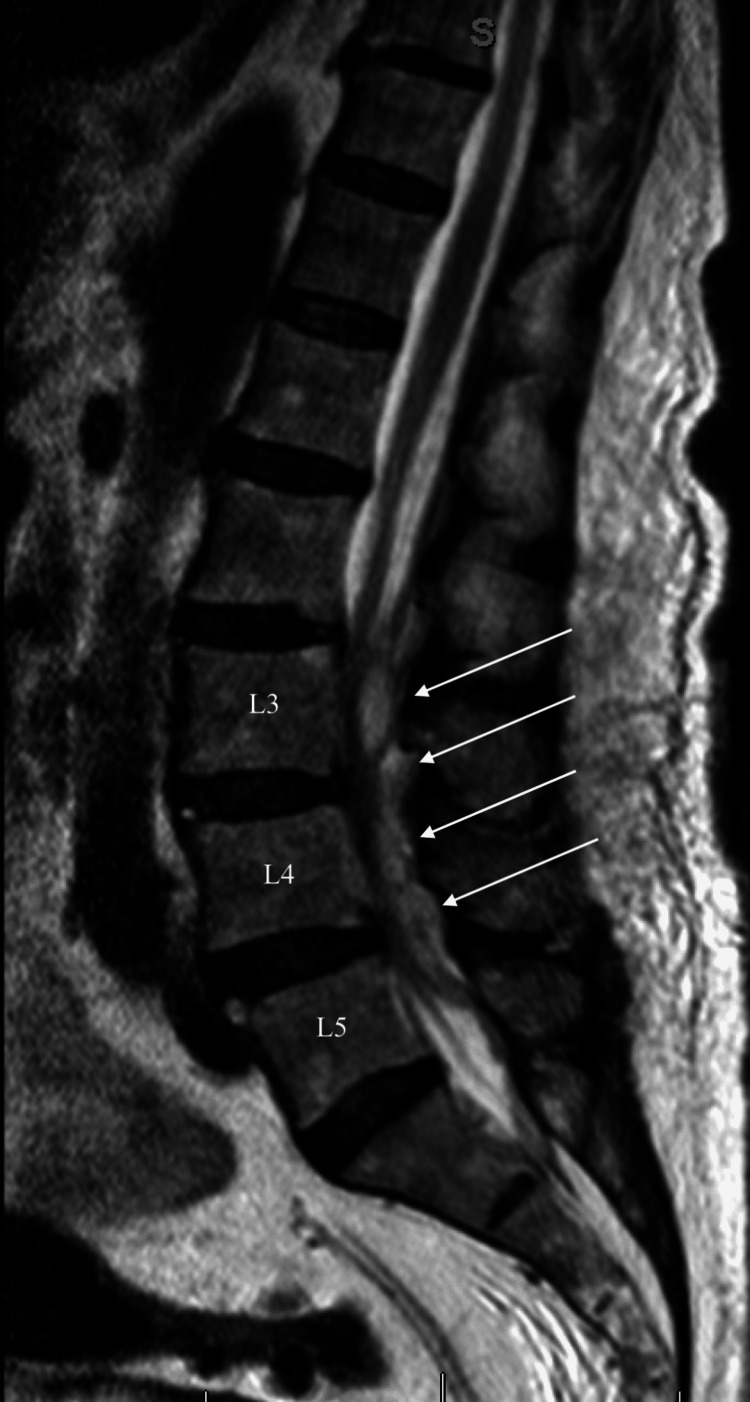
T2-weighted sagittal MRI of the lumbar spine without contrast Neurological exam findings: cranial nerves 2-12 intact without facial asymmetry. Upper extremity strength 5/5, lower extremity strength 3/5, deep tendon reflexes +2 in all extremities, no saddle anesthesia, no Hoffmann sign, no dysdiadochokinesia, and no clonus. Positive straight leg raising on the right. White Arrows: There is marked central stenosis with nerve root clumping, as well as marked facet and ligamentum flavum hypertrophy. L3: Demonstrates spondylosis, disc bulging, and slight anterolisthesis of L3 relative to L4. Additionally, there is moderate bilateral neural foraminal stenosis. L4: Demonstrates slight anterolisthesis of L4 relative to L5, along with disc bulging, facet and ligamentum flavum hypertrophy, and moderate central stenosis. There is also moderate bilateral neural foraminal stenosis. L5: Demonstrates minimal noncompressive disc bulging. In addition, there is mild facet and ligamentum flavum hypertrophy. There is no substantial central or neural foraminal stenosis. The distal cord and conus are normal.

Her elevated inflammatory markers supported the hypothesis for an ascending urinary tract infection that may have progressed into bacteremia, hence the indolent course of her infection. However, the lack of supporting evidence from her genitourinary exam findings, as well as her urine and blood cultures, raised the need to continue a broad differential diagnosis. In reconciling her neurological symptoms as a consequence of mechanical spinal cord compromise, an MRI without contrast of the lumbar spine was obtained to follow up on her CT findings (Figure 2). The imaging revealed multilevel lumbar spondylosis, degenerative disc disease, spondylolisthesis of L3-L4, ligamentum flavum hypertrophy, moderate central stenosis of L3-L5, and no distal cord compromise.

These findings were consistent with her posture-dependent pain, lower extremity weakness, and positive straight leg raising, suggesting a mechanical source of her symptoms superimposed on her indolent infection. However, they did not explain her sudden onset of weakness, which opposes the chronic, progressive nature of degenerative spinal changes. Conditions such as vertebral compression fractures or disc herniations are more classically associated with acute low back pain, yet the findings did not suggest such etiologies.

Then, overnight, she suddenly developed a fever of 101.7°F despite ongoing intravenous antibiotic therapy. The fever resolved spontaneously within an hour, and she began to report worsening pain in her right leg. Inspection of the right leg demonstrated swelling and tenderness. Venous ultrasound of the right femoral vein revealed a non-occlusive deep vein thrombosis (DVT), and she was continued on apixaban for DVT prophylaxis.

The sudden onset of fever was significant, and her differential diagnoses were reassessed with greater clarity. Her weakness did not follow an ascending, symmetrical pattern and was not associated with a history of gastrointestinal or respiratory tract infection, making Guillain-Barré syndrome unlikely. Myasthenia gravis and Lambert-Eaton myasthenic syndrome were unlikely, as her weakness was neither progressive nor altered by continuous activity. Multiple sclerosis was unlikely as no plaques were visualized at the level of her neurological deficits, and she denied any history of weakness. Moreover, she lacked upper or lower motor neuron signs suggestive of neural tract impairment that correlates with demyelination. Subacute combined degeneration was unlikely, as she lacked upper motor neuron or dorsal column deficits and showed no hematologic dysfunction indicative of cobalamin deficiency. A cerebrovascular accident was unlikely, as her findings were non-focal and posture-dependent. Weakness secondary to pyelonephritis and urosepsis was unlikely as she did not demonstrate clinical signs of peritoneal inflammation, costovertebral angle tenderness, or suprapubic tenderness. Moreover, urine cultures and CT of the abdomen-pelvis failed to demonstrate supporting evidence of a complicated urinary tract infection or renal calculi. Finally, nitrofurantoin-induced myopathy was unlikely, as it typically presents with muscle tenderness, fasciculations, atrophy, and/or elevated serum creatinine kinase. Since she did not demonstrate these findings, the likelihood remained low.

Therefore, in the context of bilateral lower extremity weakness, fever, leukocytosis, and low back pain, repeating MRI imaging of the spine for signs of a spinal epidural abscess was discussed with the patient. She agreed, given the increased suspicion for a spinal epidural abscess taking precedent over other possibilities. Her neurological exam was also reassessed, and it now demonstrated +1 patellar reflexes. Additionally, at this time, one blood culture began to grow methicillin-sensitive *Staphylococcus aureus* (MSSA). Infectious disease was consulted, and they recommended switching ceftriaxone and vancomycin to nafcillin, obtaining a second set of blood cultures, and performing an echocardiogram. Further inflammatory markers such as erythrocyte sedimentation rate (ESR) and C-reactive protein (CRP) were obtained as well, supporting the need for urgent advanced imaging (Table 2).

**Table 2:** Measured inflammatory markers ^1^Reference range for adult females over the age of 50 ESR -erythrocyte sedimentation rate; CRP - C-reactive protein

Lab test	Patient value	Female reference range
ESR	130 mm/hr	< 30 mm/hr^1^
CRP	7.5 mg/dL	< 0.3 mg/dL

**Figure 3:**
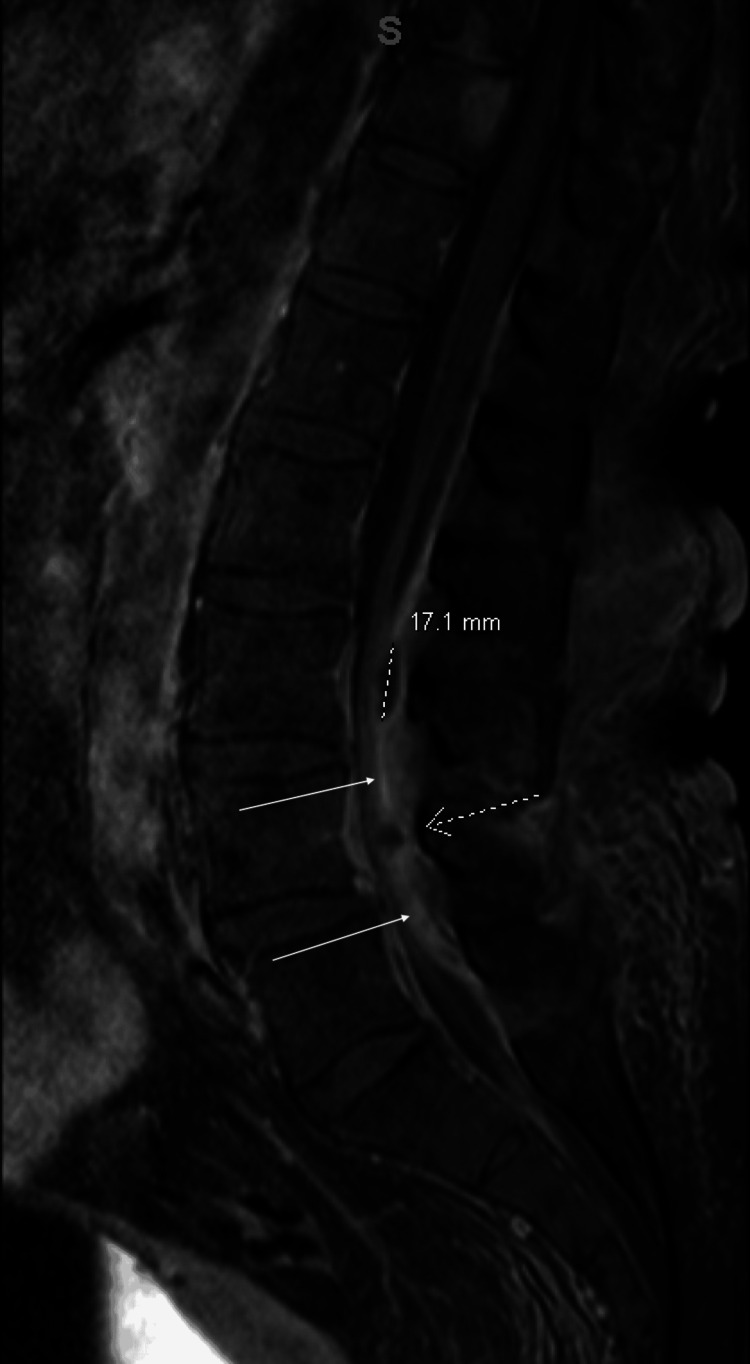
Sagittal short tau inversion recovery (STIR) MRI of the lumbar spine with contrast Key neurological exam findings: 3/5 strength and +1 patellar reflexes in bilateral lower extremities; no saddle anesthesia, Hoffmann sign, dysdiadochokinesia, or clonus. 17.1 mm: At the L3 level, there is enhancement and thickening of the dura, as well as a spinal epidural abscess measuring 17.1 mm in the cranio-caudad dimension. Dashed arrow: At the L4 level, there is a smaller fluid pocket noted within the posterior aspect of the epidural space. Solid white arrows: The enhanced dura is thickened and inflamed, causing clumping of the nerve roots and contributing to severe central stenosis.

**Figure 4:**
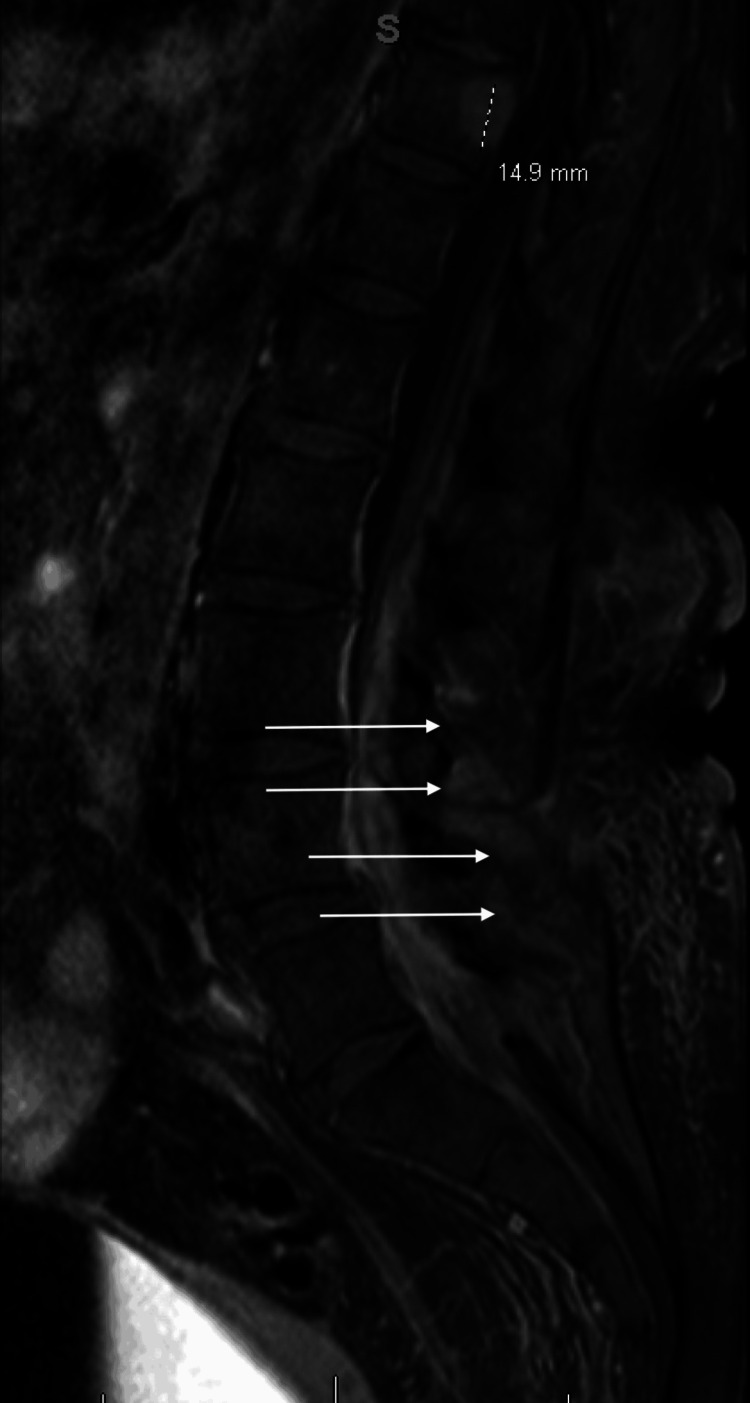
T1-weighted sagittal MRI of the lumbar spine with contrast Key neurological exam findings: 3/5 strength and +1 patellar reflexes in bilateral lower extremities; no saddle anesthesia, Hoffmann sign, dysdiadochokinesia, or clonus. 14.9 mm: At the T11 level, there is enhancement of a benign vertebral hemangioma measuring 14.9 mm in the cranio-caudad dimension, which is far removed from the dural thickening. White arrows: There is enhancement of the posterior paraspinous soft tissues, musculature, and subcutaneous tissues, consistent with myositis and cellulitis.

Her echocardiogram showed no abnormal findings; however, new findings were noted on her contrast-enhanced MRI of the lumbar spine. First, a 17.1 mm spinal epidural abscess with thickening of the dura, causing severe central stenosis, was identified (Figure 3). Second, there was enhancement of the posterior paraspinous soft tissues, musculature, and subcutaneous tissues, consistent with myositis and cellulitis (Figure 4).

**Figure 5:**
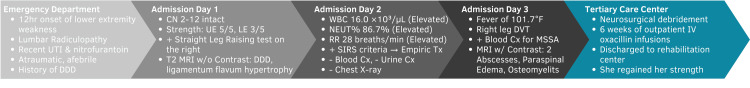
Timeline of events UTI - urinary tract infection; DDD - degenerative disc disease; CN - cranial nerves; UE - upper extremity; LE - lower extremity; W/O - without; WBC - white blood cell count; NEUT - neutrophil; RR - respiratory rate; SIRS - systemic inflammatory response syndrome; Cx - culture; DVT - deep vein thrombosis; MSSA - methicillin-sensitive *Staphylococcus aureus*; IV - intravenous

Upon confirming the diagnosis of a spinal epidural abscess, she was transferred to a tertiary care facility for neurosurgical evaluation. There, she was successfully managed with surgical debridement of the abscess in conjunction with antibiotic therapy. She responded well to the procedure and was discharged to a rehabilitation center, where she made a full recovery of motor function within two weeks. Concurrently, she also began a coordinated regimen of intravenous oxacillin for six weeks, which she received at the facility and later in an outpatient setting. Pathology results of her resected tissues later confirmed the methicillin-sensitive *Staphylococcus aureus* infection. Her only complication occurred after five weeks, when she was readmitted to the hospital due to generalized weakness and a urinary tract infection. After increasing systemic antibiotic coverage, these symptoms resolved, and she returned home. A timeline summarizing these events can be found in Figure 5.

## Discussion

A key strength of this case was the opportunity to reevaluate the patient and track changes in her condition over multiple days to ultimately arrive at the correct diagnosis. This process was facilitated by the patient’s shared decision to pursue further workup; without repeated MRI imaging, diagnostic clarity would not have been possible. It is recognized that not every patient has this level of financial freedom to weigh different diagnostic approaches [12,13]. Thus, resource stewardship is important, and potentially imprecise workup of atypical symptoms can unintentionally lead to a financial burden.

Another strength of this case is the educational value provided by the clear thought process behind the sequence of events leading up to the diagnosis and treatment of this patient [14]. By beginning with a broad differential diagnosis, many different overlapping disease mechanisms were considered. Thus, the process of systematically ruling out pertinent etiologies demonstrated how clinical reasoning is essential, especially in the context of an uncommon disease.

One limitation of this case is the lack of generalizability of the described symptoms, as the patient’s findings did not initially follow a pattern recognized in the literature [15]. However, by describing an atypical presentation, this case raises awareness of the indolent courses that spinal epidural abscesses may take.

Finally, another point of discussion is the MRI modalities utilized in this case. An MRI without contrast was initially performed due to the clinical picture being more consistent with mechanical or compressive radiculopathy, given the absence of fever, positive blood cultures, and a clear source of infection. However, as her symptoms progressed, a transient fever developed, and bacterial cultures grew *Staphylococcus aureus*; thus, the index of suspicion for an infectious etiology increased. At that point, an MRI with contrast was performed, which is more sensitive in identifying epidural abscesses, phlegmon, and dural enhancement. This repeated study revealed findings that were not appreciable on the initial non-contrast MRI. Therefore, an important takeaway for physicians and students in training is the value of obtaining an early MRI with and without contrast to minimize delays in diagnosis. After all, without the application of the most appropriate diagnostic tools, an accurate assessment can be undermined in the end.

## Conclusions

The indolent course of this spinal epidural abscess provided a diagnostic challenge in that its symptoms appeared primarily neurological before demonstrating signs of overt infection. However, in reflecting on this case, crucial teaching points can be taken away for medical students and physicians alike. That being the importance of maintaining a broad differential diagnosis, remaining vigilant against anchoring bias, conducting a thorough physical exam, considering the clinical picture in its entirety, and repeating imaging when clinically indicated.

The initial findings of radiculopathy, weakness, and spinal stenosis on imaging may have distracted from this life-threatening condition without a systematic approach. However, by considering the timeline of symptoms, risk factors, progressing neurological deficits, and evidence for infection, a spinal epidural abscess was not prematurely dismissed. For the physician-in-training, reassessing and reconciling outlying information in a consistent manner are key skills in detecting atypical disease presentations. Therefore, the index of suspicion for a spinal epidural abscess must be raised throughout the process of evaluating patients who present with signs of sudden spinal cord compression, especially in the absence of a preliminary fever.
